# Silver(I) Coordination Polymer Ligated by Bipyrazole Me_4_bpzH_2_, [Ag(X)(Me_4_bpzH_2_)] (X = CF_3_CO_2_^−^ and CF_3_SO_3_^−^, Me_4_bpzH_2_ = 3,3′,5,5′-Tetramethyl-4,4′-bipyrazole): Anion Dependent Structures and Photoluminescence Properties

**DOI:** 10.3390/molecules28072936

**Published:** 2023-03-24

**Authors:** Kiyoshi Fujisawa, Yui Kobayashi, Mitsuki Okano, Ryota Iwabuchi, Shiori Kondo, David James Young

**Affiliations:** 1 Department of Chemistry, Ibaraki University, Mito 310-8512, Ibaraki, Japan; 2 Faculty of Science and Technology, Charles Darwin University, Darwin, NT 0909, Australia; david.young@cdu.edu.au

**Keywords:** coordination polymer, silver, crystal structure, bipyrazole ligand, photoluminescence

## Abstract

Coordination polymers of transition metal ions are fascinating and important to coordination chemistry. One of the ligands known to form particularly interesting coordination polymers is 3,3′,5,5′-tetramethyl-4,4′-bipyrazole (Me_4_bpzH_2_). Group 11 metal(I) ion coordination polymers, other than those of copper(I), are relatively easy to handle because of their low reactivity towards dioxygen and moisture. However, the known silver(I) coordination polymers often have poor solubility in common solvents and so cannot be easily analyzed in solution. By using a tetramethyl substituted bipyrazole ligand, we have synthesized more soluble silver(I) complexes that contain the trifluoromethyl group in the coordinated ions CF_3_CO_2_^−^ and CF_3_SO_3_^−^ in **[Ag(CF_3_CO_2_)(Me_4_bpzH_2_)]** and **[Ag(CF_3_SO_3_)(Me_4_bpzH_2_)]**. We determined both structures by single-crystal X-ray analysis at low temperatures and compared them in detail. Moreover, we investigated the solution behavior of these coordination polymers by ^1^H-NMR, IR, Raman, UV–Vis spectroscopies, and their low-temperature, solid-state photoluminescence. The high-energy band at ~330 nm corresponded to ligand-centered (bipyrazole) fluorescence, and the low-energy band at ~400 nm to ligand-centered phosphorescence resulting from the heavy atom effect.

## 1. Introduction

**Figure 1:**
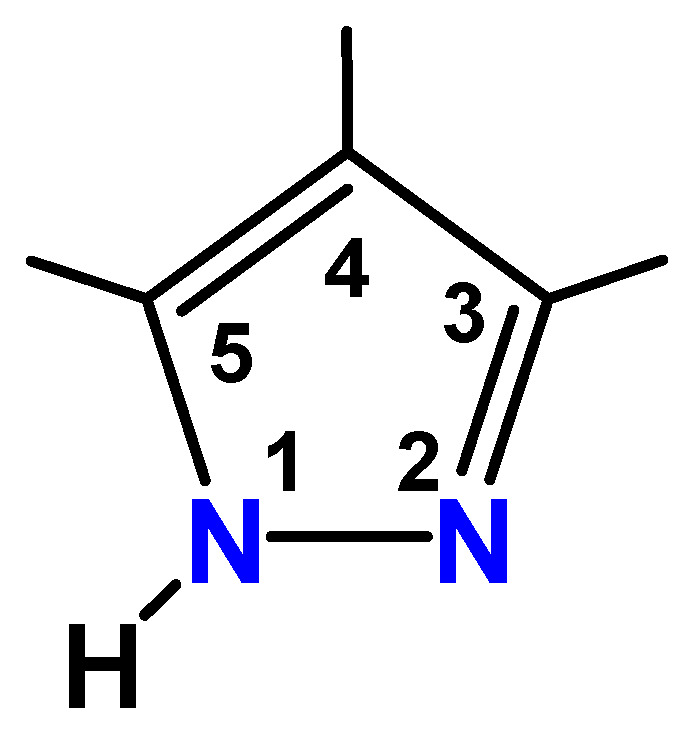
Pyrazole ligand with numbering.

Cyclic trinuclear complexes (CTCs) with coinage metal(I) ions are of theoretical and practical interest to inorganic and coordination chemists [1,2,3]. A useful class of ligands for the formation of these CTCs is pyrazolate, which is known to act as a linking ligand. The simple, neutral 1*H*-pyrazoles and their deprotonated pyrazolate anions have two adjoining nitrogen donors in the five-membered aromatic rings; thus, they can coordinate and bridge metal ions with an Npz–M–Npz linear coordination mode (pz = pyrazolate anion, C_3_H_3_N_2_^−^) [4,5,6,7]. Many substituents have been introduced at the three, four, and five positions of the five-membered ring (Figure 1).

We have been interested in modeling the structure and function of transition metal-containing proteins [8]. The active sites of some copper-containing proteins have been investigated by X-ray structural analysis, which revealed N_2_S and N_3_ donor ligands coordinating to the metal center [9]. We similarly used N_3_ tripodal ligands in which three pyrazoles linked by a boron atom in hydridotris(pyrazolyl)borate gave copper(II) dioxygen complexes as simple hemocyanin models [8,10,11] and copper(II) thiolato complexes for copper-containing electron transfer model complexes [12]. As part of these investigations, we made numerous pyrazoles, varying in their steric and electronic properties. In the present work, we have explored the use of pyrazole to make new CTC compounds.

**Figure 2:**
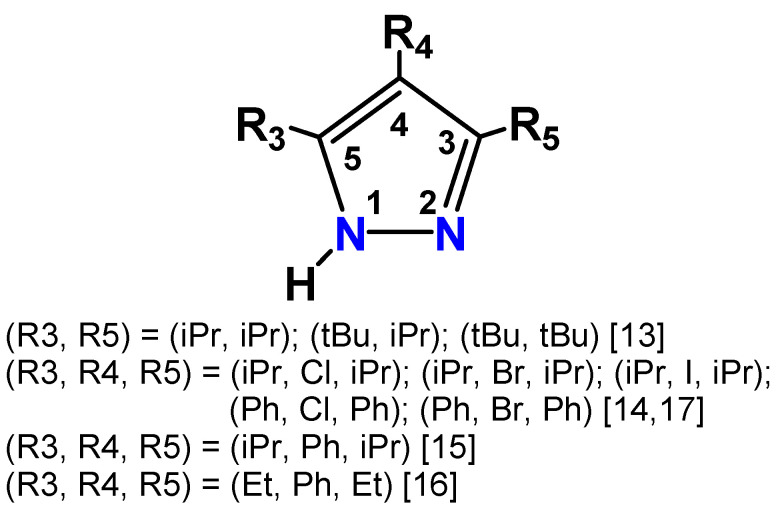
Various pyrazoles used to make silver(I) CTCs [13,14,15,16,17].

Our first publication reported silver(I) CTCs with 3,5-diisopropyl, 3-isopropyl-5-tertiary butyl, and 3,5-ditertiary butyl pyrazoles (Figure 2). We showed that the geometries of these complexes were greatly influenced by the steric influence exerted by the substituent groups on the pyrazolyl rings, and the differences in the central metal(I) ionic radius in trinuclear complexes [Ag(μ-3,5-iPr_2_pz)]_3_, [Ag(μ-3-tBu-5-iPrpz)]_3_, and tetranuclear [Ag(μ-3,5-tBu_2_pz)]_4_ [13]. Halogen atoms were introduced using *N*-halosuccinimides, and the electronegativity of the halogen substituent could be correlated with the strength of the Ag⋯Ag interaction and the wavelength of solid-state photoluminescence in dimeric trinuclear (hexanuclear) complexes {[Ag(μ-4-X-3,5-R_2_pz)]_3_}_2_ (R = iPr, X = Cl, Br, and I) and trinuclear [Ag(μ-4-X-3,5-R_2_pz)]_3_ (R = iPr, X = I; R = Ph, X = Cl, R = Ph, X = Br) [14]. Phenyl substituents in [Ag(μ-4-Ph-3,5-iPr_2_pz)]_3_ altered the solid-state crystal packing to a stair-type structure, which was quite distinct from that observed for the parent [Ag(μ-3,5-iPr_2_pz)]_3_ [15]. Employing the less hindered ethyl group gave a dimeric trinuclear (hexanuclear) complex with two intermolecular argentophilic interactions {[Ag(μ-4-Ph-3,5-Et_2_pz)]_3_}_2_ [16]. This complex easily incorporated aromatic guests to form arene-sandwiched, π acid/base complexes, [Ag(μ-4-Ph-3,5-Et_2_pz)]_3_(toluene), and [Ag(μ-4-Ph-3,5-Et_2_pz)]_3_(mesitylene). An unexpected synthetic outcome yielded a silver(I) coordination polymer [Ag(µ-4-Cl-3,5-iPr_2_pz)]*_n_* from the reaction of {[Ag(µ-4-Cl-3,5-iPr_2_pz)]}_2_ with (^n^Bu_4_N)[Ag(CN)_2_] [17]. We have expanded this study to make a silver(I) coordination polymer with 3,3′,5,5′-tetramethyl-4,4′-bipyrazole (Me_4_bpzH_2_).

**Figure 3:**
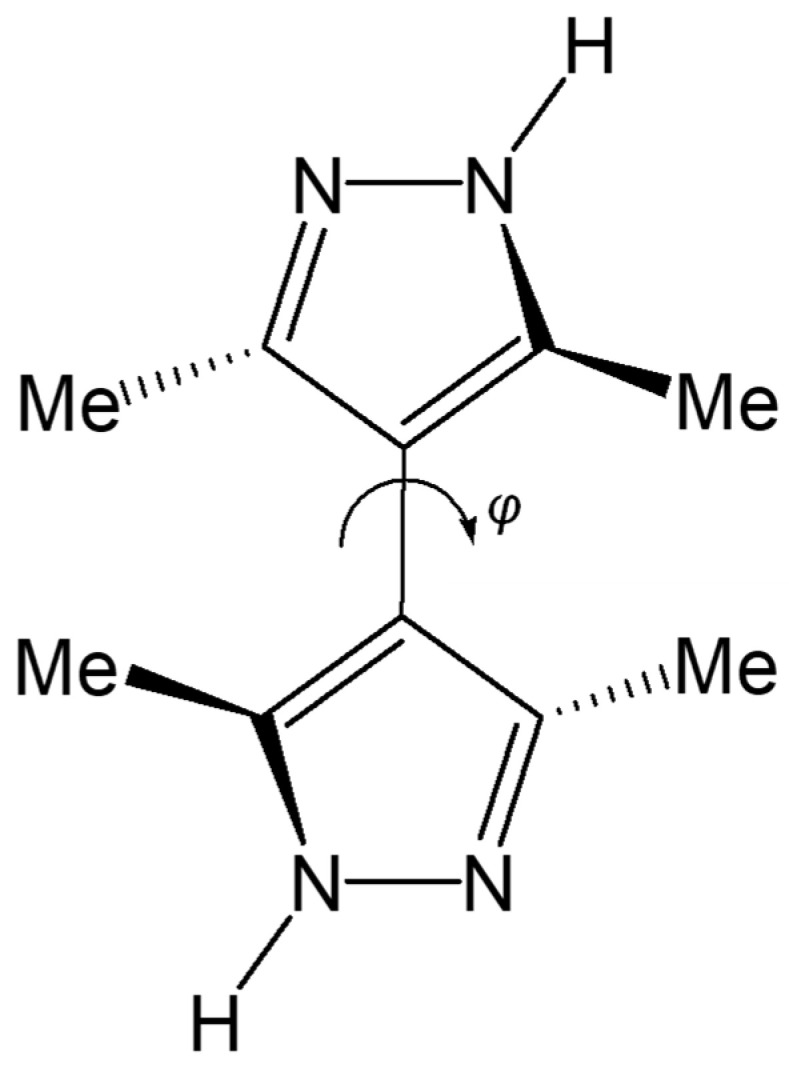
3,3′,5,5′-Tetramethyl-4,4′-bipyrazole (Me_4_bpzH_2_) and the interplanar angle (*φ*) of bipyrazole.

Many transition metals ligated by 3,3′,5,5′-tetramethyl-4,4′-bipyrazole (Me_4_bpzH_2_) have been reported [18]. The geometry of this bipyrazole is presumably controlled by the steric repulsion of the four-methyl groups, which influence the configuration of the two pyrazole rings and interplanar angle (*φ*), which is also controlled by the metal ion and its coordination environment (Figure 3). Single-crystal structures reported for silver(I) complexes ligated by bipyrazoles include the following: [Ag(NO_3_)(Me_4_bpzH_2_)]·MeOH [19], [Ag(Me_4_bpzH_2_)](ClO_4_) [20], [Ag(Me_4_bpzH_2_)](PO_2_F_2_) [20], [Ag_4_(NO_3_)_4_(Me_4_bpzH_2_)_5_]·2H_2_O [20], [Ag(CF_3_SO_3_)(Me_4_bpzH_2_)] [20], [Ag_2_(CF_3_CO_2_)_2_(Me_4_bpzH_2_)_3_] [20], [Ag(C_2_F_5_CO_2_)(Me_4_bpzH_2_)] [20], [Ag_2_(Me_4_bpz)] [21,22], [Ag_30_(Me_4_bpz)_15_]·10(C_6_H_6_) [21,22], [Ag_30_(Me_4_bpz)_15_]·9(C_6_H_5_CH_3_) [21,22], [Ag(*p*-HO_2_C_6_H_4_CO_2_)(Me_4_bpzH_2_)] [23], [Ag_2_(*m*-O_2_C_6_H_4_CO_2_)(Me_4_bpzH_2_)_2_] [23], [Ag(CH_3_CO_2_)(Me_4_bpzH_2_)]·5.4H_2_O [23], [Ag_6_(Ph_4_bpz)_3_] (Ph_2_bpz = 3,3′,5,5′-tetraphenyl-4,4′-bipyrazole dianion) [24], and [Ag_2_(SO_4_)(Me_4_bpzH_2_)_2_]·3H_2_O [25]. Depending on the metal-to-ligand ratio, and other factors, it is possible to form many structures, such as coordination polymers with trinuclear structures. However, silver(I) coordination polymers are insoluble in most solvents once formed. To overcome this disadvantage, anions with trifluoromethyl groups such as CF_3_CO_2_^−^ and CF_3_SO_3_^−^ were used in the present study as coordinated ions. We have previously reported the use of the trifluoromethyl group to make the manganese(II) complex [Mn^II^{HB(3-CF_3_-5-Mepz)_3_}_2_], where HB(3-CF_3_-5-Mepz)_3_^−^ = hydridotris(3-trifluoromethyl-5-methylpyrazolyl-1-yl)borate anion [26] and copper(I) complexes [Cu^I^{HB(3-CF_3_-5-Mepz)_3_}(CO)] and [Cu^I^{HB(3-CF_3_-5-Mepz)_3_}(PPh_3_)] [27]. The trifluoromethyl group has unique electronegativity, hydrophobicity, metabolic stability, and bioavailability. It is therefore widely employed in medicine, agrochemicals, and organic materials [28]. In the present work, we report the synthesis of silver(I) coordination polymers, **[Ag(CF_3_CO_2_)(Me_4_bpzH_2_)]** and **[Ag(CF_3_SO_3_)(Me_4_bpzH_2_)]**, and their characterization by ^1^H-NMR, IR, Raman, UV–Vis, and photoluminescence spectroscopies. The reported structure of **[Ag(CF_3_SO_3_)(Me_4_bpzH_2_)]** [20] had a severe disorder in the trifluoromethyl groups, and this problem was avoided in the present study by acquiring the diffraction data at −95 °C.

## 2. Results and Discussion

### 2.1. Synthesis

**Figure 4:**
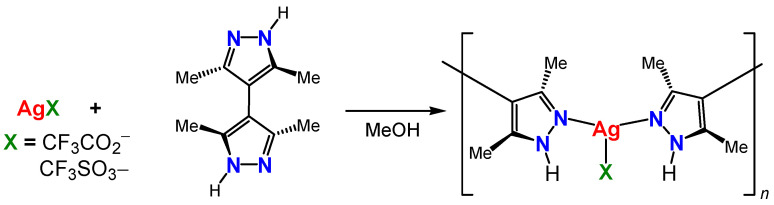
Synthesis of silver(I) coordination polymers **[Ag(X)(Me_4_bpzH_2_)]**, X = CF_3_CO_2_^–^, and CF_3_SO_3_^–^.

The reactions of 3,3′,5,5′-tetramethyl-4,4′-bipyrazole (Me_4_bpzH_2_) [19,29] with one equivalent of silver(I) ions, Ag(CF_3_CO_2_) and Ag(CF_3_SO_3_), were carried out at room temperature (Figure 4), and they were given white powders after 48 h. The yields were modest (50–60%). Single crystals were obtained from the filtrate by slow evaporation at room temperature.

Powder X-ray diffraction analysis of the white powders matched the single-crystal structures, indicating phase purity (Figures S1 and S2 from Supplementary Materials).

### 2.2. Structures

**Figure 5:**
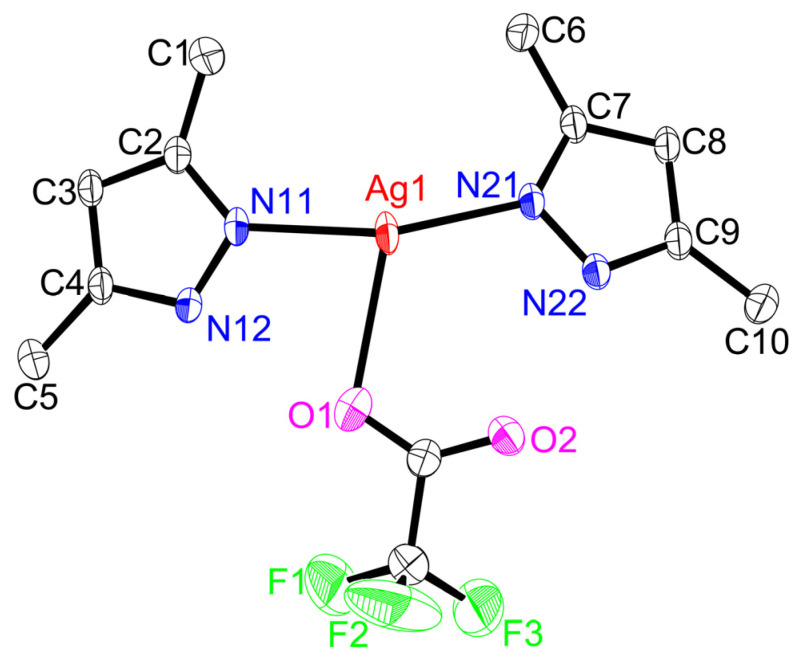
Crystal structure of **[Ag(CF_3_CO_2_)(Me_4_bpzH_2_)]** (silver(I) core) showing 50% displacement ellipsoids and the atom labeling scheme. Hydrogen atoms are omitted for clarity. Relevant bond lengths (Å) and angles (°): Ag1–N11, 2.143(2); Ag1–N21, 2.127(2); Ag1–O1, 2.544(2); Ag1···O2, 3.349(2); O1–Ag1–N11, 81.51(8); O1–Ag1–N21, 112.54(8); N11–Ag1–N21, 158.49(9), O2···Ag1–N11, 123.29(7); and O2···Ag1–N21, 72.96(7).

**Figure 6:**
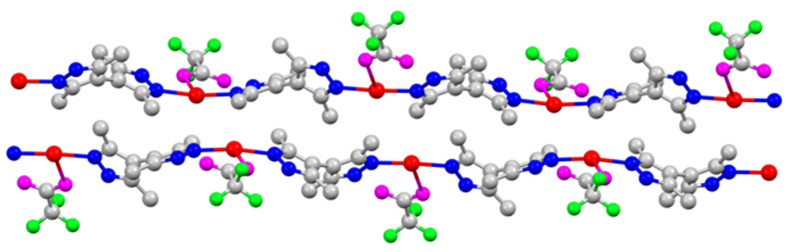
The 1-D polynuclear structure of **[Ag(CF_3_CO_2_)(Me_4_bpzH_2_)]**. Hydrogen atoms are omitted for clarity. Color: silver: red, nitrogen: blue, oxygen: magenta, fluorine: green, and carbon: gray.

**Figure 7:**
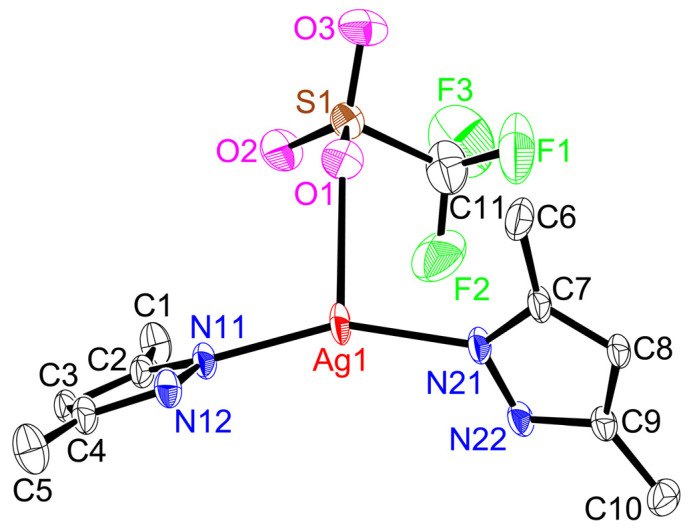
Crystal structure of **[Ag(CF_3_SO_3_)(Me_4_bpzH_2_)]** (silver(I) core) showing 50% displacement ellipsoids and the atom labeling scheme. Hydrogen atoms are omitted for clarity. Relevant bond lengths (Å) and angles (°): Ag1–N11, 2.145(3); Ag1–N21, 2.146(3); Ag1–O1, 2.678(3); Ag1···O2, 4.233(2); O1–Ag1–N11, 105.53(9); O1–Ag1–N21, 98.36(10); N11–Ag1–N21, 155.61(11), O2···Ag1–N11, 88.92(7); and O2···Ag1–N21, 109.53(8).

**Figure 8:**
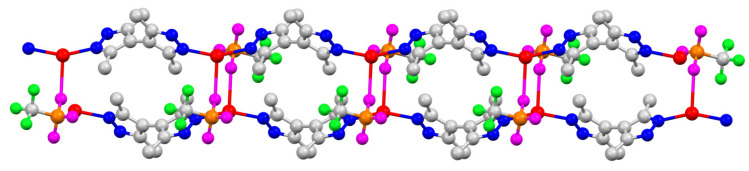
The 1-D polynuclear structure of **[Ag(CF_3_SO_3_)(Me_4_bpzH_2_)]**. Hydrogen atoms are omitted for clarity. Color: silver: red, nitrogen: blue, oxygen: magenta, sulfur: orange, fluorine: green, and carbon: gray.

**Figure 9:**
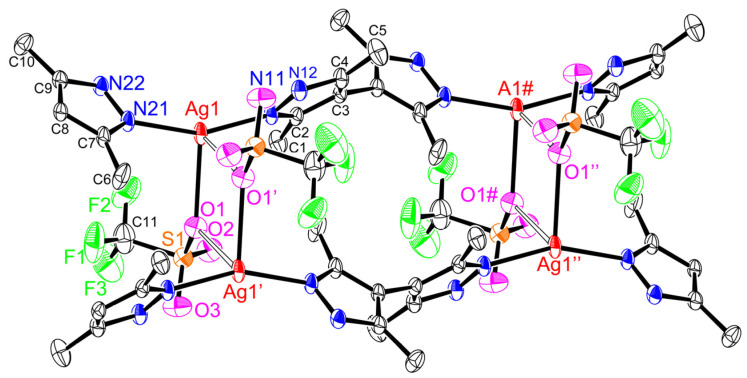
Fragments of double-chain structures of **[Ag(CF_3_SO_3_)(Me_4_bpzH_2_)]** showing 50% displacement ellipsoids and the atom labeling scheme. Hydrogen atoms are omitted for clarity. Relevant bond lengths (Å): Ag1–O1, 2.678(3); Ag1···O1′, 2.822(2); Ag1···Ag1′, 4.4592(4). Symmetry operators: ′: –x + 1, –Y + 1, –Z + 2; ″: –x + 1, –Y + 2, –Z + 2; #: X, Y + 1, Z.

Single-crystal X-ray structures of coordination polymers (Figure 5, Figure 6, Figure 7, Figure 8 and Figure 9), **[Ag(CF_3_CO_2_)(Me_4_bpzH_2_)]** and **[Ag(CF_3_SO_3_)(Me_4_bpzH_2_)],** are shown in Figure 5 and Figure 7, respectively. The 1-D polynuclear structures of **[Ag(CF_3_CO_2_)(Me_4_bpzH_2_)]** and **[Ag(CF_3_SO_3_)(Me_4_bpzH_2_)]** are presented in Figure 6 and Figure 8, respectively. Fragments of the double-chain structures of **[Ag(CF_3_SO_3_)(Me_4_bpzH_2_)]** are shown in Figure 9.

The Ag(I) atoms in **[Ag(CF_3_CO_2_)(Me_4_bpzH_2_)]** (Figure 5) were coordinated by two pyrazole N atoms of two Me_4_bpzH_2_ and one O atom of a CF_3_CO_2_^−^ anion, giving a distorted trigonal pyramidal geometry with 0.27 Å distance between the Ag(I) ion and the plane created by the coordinated atoms of the N_2_O ligand donor set. The coordinated pyrazoles’ dihedral angle in Me_4_bpzH_2_ was 42.9°, and the shortest Ag⋯Ag distance was 9.9857(4) Å. The dihedral angle of the bipyrazole (*φ*) in Figure 3 is 62.7°, which is within the range of the reported values. Therefore, in the 1-D polynuclear structure, a zig-zag configuration was formed (Figure 6). Likewise, the coordinated CF_3_CO_2_^−^ anions were also located in a zig-zag pattern. The distance to the next Ag(I) ion was 18.5775(4) Å, and the dihedral angle between these pyrazoles was 0°. The carboxylate oxygen was coordinated to the Ag(I) ions at a relatively long distance of Ag1–O1, 2.544(2) Å with a very weak Ag1···O2 interaction of 3.349(2) Å. This conformation was stabilized by two intramolecular hydrogen bonds of 2.801(3) Å N12⋯O1 and 2.738(3) N22⋯O2. The interdimer Ag⋯Ag distances were 3.4250(4) and 8.6779(3) Å (Figure 6). The former is almost the same as the sum of twice Bondi’s van der Waals radius (3.44 Å) [30], indicating small argentophilic interactions [31].

The Ag(I) atoms in **[Ag(CF_3_SO_3_)(Me_4_bpzH_2_)]** (Figure 7) were coordinated by two pyrazole N atoms of two Me_4_bpzH_2_ and one O atom of the CF_3_SO_3_^−^ anion, giving a distorted trigonal pyramidal geometry with 0.08 Å in distance between the Ag(I) ion and the plane created by the coordinated atoms. The coordinated pyrazoles’ dihedral angle in Me_4_bpzH_2_ was 77.05°, and the shortest Ag⋯Ag distance was 9.9158(4) Å. The dihedral angle of the bipyrazole (*φ*) was 77.05° (Figure 3), which is in the range of the reported values. Therefore, in the 1-D polynuclear structure, a linear configuration was formed (Figure 8). The coordinated anions CF_3_SO_3_^−^ were oriented in the same direction. The distance to the next Ag(I) ion was 19.815(5) Å, this value is twice the Ag1···Ag1 distance of 9.9158(4) Å, so that each Ag(I) ion was linear. The dihedral angle between these pyrazoles was 0°. The carboxylate oxygen was coordinated to the Ag(I) ions at a relatively long distance of Ag1···O1, 2.678(3) Å with no interaction between Ag1···O2, 4.233(2) Å. This conformation was stabilized by two intermolecular hydrogen bonds of 2.844(4) Å N12⋯O3 and 2.865(4) N22⋯O2. Moreover, the interdimer Ag⋯Ag distance was 4.4592(4), which is longer than the sum of twice the Bondi’s van der Waals radius (3.44 Å) [30], indicating almost no argentophilic interaction [31] (Figure 9). However, **[Ag(CF_3_SO_3_)(Me_4_bpzH_2_)]** forms a double-chain structure (Figure 9).

### 2.3. Solution-State Properties

The ^1^H-NMR spectrum of the obtained white powder **[Ag(CF_3_SO_3_)(Me_4_bpzH_2_)]** in CDCl_3_ revealed only a broad 1.61 ppm signal (Figure S3 from Supplementary Materials), which was different from that of the ligand, Me_4_bpzH_2_ at 2.10 ppm (Figure S4 from Supplementary Materials). This observation is also supported by its solution-state UV–Vis spectra in MeOH (Figure S5 from Supplementary Materials). A broad absorption of Me_4_bpzH_2_ in the UV region was observed at around 230 nm, and the shoulder of **[Ag(CF_3_SO_3_)(Me_4_bpzH_2_)]** was observed at almost the same energy, but with a different molecular extinction coefficient. Therefore, the structure of **[Ag(CF_3_SO_3_)(Me_4_bpzH_2_)]** in the solution remains intact. However, we did not measure concentration dependences in the NMR or UV–Vis experiments. Unfortunately, the solubility of **[Ag(CF_3_CO_2_)(Me_4_bpzH_2_)]** was poor, and we could not obtain a UV–Vis spectrum in the MeOH solution.

### 2.4. Solid-State Properties

**Figure 10:**
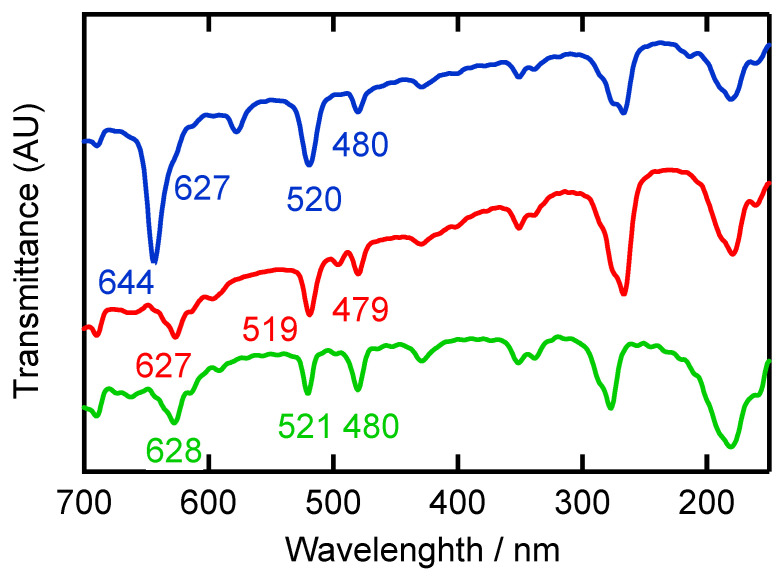
Far-IR spectra of the ligand Me_4_bpzH_2_ (green line), and silver(I) polymers **[Ag(CF_3_CO_2_)(Me_4_bpzH_2_)]** (red line), and **[Ag(CF_3_SO_3_)(Me_4_bpzH_2_)**] (blue line) at room temperature.

In **[Ag(CF_3_CO_2_)(Me_4_bpzH_2_)]**, the characteristic CO_2_ stretching vibrations could be observed in the IR region at 1683 cm^−1^, and in **[Ag(CF_3_CO_2_)(Me_4_bpzH_2_)],** the characteristic stretching vibrations from the CF_3_SO_3_^−^ group were observed at 1260 cm^−1^ ν_as_(SO_3_), 1175 cm^−1^ ν_as_(CF_3_), 1026 cm^−1^ ν_s_(SO_3_) in the IR spectrum, and 1027 cm^−1^ ν_s_(SO_3_) in the Raman spectrum [32,33]. Strong peaks in the far-IR region were assigned to the ν(C–C) of bipyrazole, which was observed at 627 cm^−1^ (IR) and 625 cm^−1^ (Raman) in **[Ag(CF_3_CO_2_)(Me_4_bpzH_2_)]**, and at 627 cm^−1^ (shoulder) (IR) and 624 cm^−1^ (Raman) in **[Ag(CF_3_SO_3_)(Me_4_bpzH_2_)]**, and at 628 cm^−1^ (IR) and 619 cm^−1^ (Raman) in Me_4_bpzH_2_. An additional peak at 644 cm^−1^ was assigned to δ_s_(SO_3_) (Figure 10, Figures S6 and S7 from Supplementary Materials).

The Ag–N stretching vibration has been previously reported at ~500 cm^−1^ [13,14,15,16,17,32]. However, the ligand Me_4_bpzH_2_ exhibited some peaks in this region. Therefore, we cannot conclusively assign this vibration as ν(Ag–N). The Ag–O stretching vibration could be assigned at 519 cm^−1^ (IR) in **[Ag(CF_3_CO_2_)(Me_4_bpzH_2_)]** and 520 cm^−1^ (IR) in **[Ag(CF_3_SO_3_)(Me_4_bpzH_2_)]**, compared with the Ag–O stretching vibration of its precursors, 518 cm^−1^ (IR) in [Ag(CF_3_CO_2_)] and 519 cm^−1^ (IR) in [Ag(CF_3_SO_3_)]. These vibration data confirm the solid-state structure observed by X-ray crystallography.

The emission spectra of the silver(I) complexes **[Ag(CF_3_CO_2_)(Me_4_bpzH_2_)]**, **[Ag(CF_3_SO_3_)(Me_4_bpzH_2_)**], and Me_4_bpzH_2_ are shown in Figure S8 from Supplementary Materials (solid-state and solution-state at 298 K), Figure S9 from Supplementary Materials (temperature dependence, Me_4_bpzH_2_), Figure S10 from Supplementary Materials (temperature dependence, **[Ag(CF_3_CO_2_)(Me_4_bpzH_2_)]**), Figure S11 from Supplementary Materials (temperature dependence, **[Ag(CF_3_SO_3_)(Me_4_bpzH_2_)**]), Figure S12 from Supplementary Materials (solid-state at 173 K, comparison), and Figure S13 from Supplementary Materials (solid-state at 298 K, comparison).

**Figure 11:**
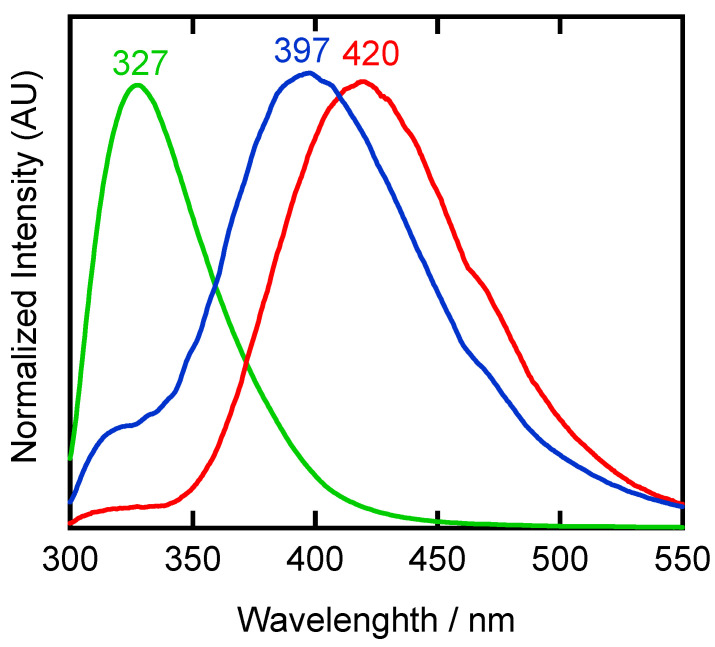
Solid-state photoluminescence spectra of ligand Me_4_bpzH_2_ (green line, 250 nm excitation), and silver(I) polymers **[Ag(CF_3_CO_2_)(Me_4_bpzH_2_)],** (red line, 250 nm excitation), and **[Ag(CF_3_SO_3_)(Me_4_bpzH_2_)]** (blue line, 240 nm excitation) at 83 K.

At 298 K, there were no significant differences between silver(I) complexes **[Ag(CF_3_CO_2_)(Me_4_bpzH_2_)]** and **[Ag(CF_3_SO_3_)(Me_4_bpzH_2_)**] and the ligand Me_4_bpzH_2_. However, some shift was observed between the solid-state and solution-state spectra of **[Ag(CF_3_SO_3_)(Me_4_bpzH_2_)**] (Figures S8 and S13 from Supplementary Materials). This may be caused by the dissociation of **[Ag(CF_3_SO_3_)(Me_4_bpzH_2_)**] in the solution. At lower temperatures of 173 K and 83 K, a new broad emission band was observed at 420 nm in **[Ag(CF_3_CO_2_)(Me_4_bpzH_2_)]** and at 397 nm in **[Ag(CF_3_SO_3_)(Me_4_bpzH_2_)**] (Figure 11, Figure S10–S12 from Supplementary Materials).

In addition to the most intense 420 nm emission band of **[Ag(CF_3_CO_2_)(Me_4_bpzH_2_)]** and the 397 nm emission of **[Ag(CF_3_SO_3_)(Me_4_bpzH_2_)],** the corresponding measurements at 83 K revealed an additional band around ~330 nm, which was also observed in the ligand Me_4_bpzH_2_ at the same temperature (Figure 11). This higher energy emission band may be from ligand-based phosphorescence [25]. The lower energy emission band was attributed to metal-based phosphorescence arising from closed shell d^10^–d^10^ intermolecular argentophilic (Ag···Ag) interactions [13,14,15,16,17,34,35,36]. Both ~330 nm and ~400 nm bands were ascribed to ligand-based phosphorescence, since **[Ag(CF_3_SO_3_)(Me_4_bpzH_2_)]** has no argentophilic interaction, as indicated by the interdimer Ag⋯Ag distance of 4.4592(4) Å. The latter emission was also attributed to the heavy metal effect [1,2,3]. This explanation has been proposed based on experimental observations of the previously reported [Ag_2_(SO_4_)(Me_4_bpzH_2_)_2_]·3H_2_O [25]. We are now in the process of probing the origin of this behavior through theoretical and more detailed physicochemical research.

## 3. Materials and Methods

### 3.1. Material and General Techniques

The preparation and handling of the two silver(I) complexes were performed under an argon atmosphere using standard Schlenk tube techniques under light-shielded conditions. Ultra-dry methanol was purchased from Wako Pure Chemical Ind. Ltd. and deoxygenated by purging with argon gas. Deuteriochloroform was obtained from Cambridge Isotope Laboratories, Inc. Other reagents were commercially available and used without further purification. The 3,3′,5,5′-tetramethyl-4,4′-bipyrazole (Me_4_bpzH_2_) was prepared by published methods [19,28]. The purity of the ligand was checked by ^1^H-NMR spectroscopy.

### 3.2. Instrumentation

IR spectra (4000–400 cm^−1^) and far-IR spectra (680–150 cm^−1^) were recorded as KBr pellets using a JASCO FT/IR-6300 spectrophotometer under ambient conditions (JASCO, Tokyo, Japan) and as CsI pellets using a JASCO FT/IR 6700 spectrophotometer under vacuum (JASCO, Tokyo, Japan), respectively. Raman spectra (4000–200 cm^−1^) were measured as powders on a JASCO RFT600 spectrophotometer with a YAG laser 600 mW (JASCO, Tokyo, Japan). Abbreviations used in the description of vibration data are as follows: s, strong; m, medium; and w, weak. ^1^H-NMR (500 MHz) and ^13^C-NMR spectra (125 MHz) were obtained on a Bruker AVANCE III-500 NMR spectrometer at room temperature (298 K) in CDCl_3_-*d*_1_ or CD_3_OD-*d*_3_ (Bruker Japan, Yokohama, Japan). ^1^H and ^13^C chemical shifts were reported as *δ* values relative to residual solvent peaks. UV–Vis spectra (solution and solid, 1000–200 nm) were recorded on a JASCO V-570 spectrophotometer (JASCO, Tokyo, Japan). The values of *ε* were calculated per silver(I) ion. Solid samples (mulls) for UV–Vis spectroscopy were prepared by finely grinding microcrystalline material into powders with a mortar and pestle and then adding mulling agents (nujol, poly(dimethylsiloxane), viscosity 10,000) (Aldrich)) before uniformly spreading between quartz plates. Luminescence spectra were recorded on a JASCO FP-6500 (solution and solid, 600–300 nm) spectrofluorometer (JASCO, Tokyo, Japan). Low-temperature luminescence spectra were recorded using solid samples, which were prepared by finely grinding microcrystalline material into powders with a mortar between quartz plates cooled with a liquid nitrogen cryostat (CoolSpeK USP-203) from Unisoku Scientific Instruments (Osaka, Japan). Powder X-ray diffraction (XRD) measurements were conducted on a Rigaku SmartLab-SP/IUA X-ray diffractometer (Rigaku, Tokyo, Japan) with a Cu K*α* radiation (*λ* = 1.54 Å) source (40 kV, 30 mA) and a high-speed one-dimensional detector D/teX Ultra 250. The 2*θ* was measured in the range of 5–90° with a scan step of 0.02° and scan speed of 10° min^−1^. Solid samples for XRD were prepared by finely grinding microcrystalline materials into powders with a mortar and pestle and then placing them on an aluminum dish (0.2 mm thickness). Simulated powdered XRD patterns were calculated from single-crystal data using the MERCURY software suite from CCDC. The elemental analyses (C, H, and N) were performed by the Chemical Analysis Center of Ibaraki University.

### 3.3. Preparation of Ligand and Complexes

- 3,3′,5,5′-Tetramethyl-4,4′-bipyrazole (Me_4_bpzH_2_)

The bispyrazole ligand was prepared by published methods [19,28]. The purity of the ligand was checked by ^1^H-NMR spectroscopy and characterized as indicated below.

Calcd for C_10_H_16_N_4_O = Me_4_bpzH_2_•H_2_O: C, 57.67; H, 7.74; N, 26.90. Found: C, 57.95; H, 7.82; N 27.13. ^1^H-NMR (CDCl_3_, 500 MHz): *δ*/ppm (assignments): 2.18 (s, 12 H, Me). ^1^H-NMR (CD_3_OD, 500 MHz): *δ*/ppm (assignments): 2.05 (s, br, 12 H, Me). ^13^C-NMR (CD_3_OD, 125 MHz): *δ*/ppm (assignments): 9.8 (3- or 5-Me), 12.2 (3- or 5-Me), 109.8 (pz-4*C*), 139.6 (3- or 5-pz*C*), 149.4 (3- or 5-pz*C*). IR (KBr, cm^−1^): 3200 s ν(N–H), 3082 s ν(N–H), 2925 s ν(C–H), 2824 s ν(C–H), 1614 w, 1568 m, 1545 m, 1416 s, 1371 w, 1309 m, 1291 m, 1256 m, 1172 w, 1062 w, 1041 w, 1016 s, 842 m, 786 s, 625 w, 519 w, 479 w. Far–IR (CsI, cm^−1^): 662 w, 628 s ν(C–C), 591 w, 521 s, 480 s, 429 m, 351 m, 338 m, 277 s, 180 s. Far–IR (CsI, cm^−1^): 662 w, 628 s ν(C–C), 591 w, 521 s, 480 s, 429 m, 351 m, 338 m, 277 s, 180 s. Raman (solid, cm^−1^): 2928 s ν(C–H), 1623 m, 1539 w, 1473 m, 1421 m, 1375 w, 1307 w, 1156 w, 1139 w, 973 w, 783 w, 710 w, 619 s ν(C–C), 592 m, 518 w, 486 w, 423 w, 343 m. UV–Vis (solution, methanol, *λ*_max_/nm (*ε*/cm^−1^ mol^−1^ dm^3^)): 223 (5100). Emission (solid, ex. 250 nm, *λ*_max_/nm): 83 K, 327; 173 K, 328, 83 K, 328.

- **[Ag(CF_3_CO_2_)(Me_4_bpzH_2_)]**

A solution of 3,3′,5,5′-tetramethyl-4,4′-bipyrazole (Me_4_bpzH_2_) (388 mg, 2.04 mmol) in methanol (10 cm^3^) was added to a solution of silver(I) trifluoroacetate (446 mg, 2.02 mmol) in methanol (10 cm^3^). The mixture was stirred for 48 h, and the resulting powder was filtered and dried under vacuum. The colorless powder was obtained by filtration (561 mg, 1.36 mmol, 67%). Colorless crystals for X-ray analysis were obtained from the filtrate.

Calcd for C_12_H_14_AgF_3_N_4_O_2_: C, 35.06; H, 3.43; N, 13.63. Found: C, 34.94; H, 3.51; N 13.67.

IR (KBr, cm^−1^): 3305 s, 3079 s, 2929 s, 1683 s ν(C=O), 1558 m, 1542 m, 1496 m, 1462 m, 1429 m, 1374 w, 1281 m, 1261 m, 1206 s, 1132 s, 1042 m, 835 m, 798 m, 780 m, 720 m, 708 m, 616 w, 597 w, 566 w. Far–IR (CsI, cm^−1^): 627 s ν(C–C), 597 w, 519 s ν(Ag–O), 496 w, 479 m ν(Ag–N), 429 w, 351 m, 266 s, 179 s. Raman (solid, cm^−1^): 2970 m ν(C–H), 2932 s ν(C–H), 1619 s ν(C=O), 1544 w, 1489 m, 1450 m, 1428 s, 1388 m, 1303 w, 1188 w, 835 w, 625 s ν(C–C), 592 w, 533 w, 454 m ν(Ag–N), 412 w, 349 w, 300 w. Emission (solid, ex. 250 nm, λ_max_/nm): 83 K, 420; 173 K, 423; 298 K, 331.

- **[Ag(CF_3_SO_3_)(Me_4_bpzH_2_)]**

A solution of 3,3′,5,5′-tetramethyl-4,4′-bipyrazole (Me_4_bpzH_2_) (271 mg, 1.43 mmol) in methanol (10 cm^3^) was added to a solution of silver(I) trifluoromethanesulfonate (366 mg, 1.43 mmol) in methanol (10 cm^3^). The mixture was stirred for 48 h. A colorless powder was obtained (349 mg, 0.78 mmol, 55%) by slow evaporation of the transparent solution. Colorless crystals for X-ray analysis were obtained by recrystallization from methanol at room temperature.

Calcd for C_11_H_14_AgF_3_N_4_O_3_S: C, 29.54; H, 3.16; N, 12.53. Found: C, 29.52; H, 3.19; N, 12.56.

IR (KBr, cm^−1^): 3315 s, 3245 s, 3099 m, 2964 m, 2927 m, 1628 w, 1598 m, 1563 m, 1545 m, 1463 m, 1420 m, 1378 w, 1377 w, 1260 s ν_as_(SO_3_), 1227 s, 1175 s ν_as_(CF_3_), 1157 m, 1104 w, 1026 s ν_s_(SO_3_), 800 m, 784 w, 732 m, 707 w, 638 s, 576 w, 519 m. Far-IR (CsI, cm^−1^): 689 w, 644 s δ_s_(SO_3_), 627 sh ν(C–C), 596 w, 578 m, 520 s ν(Ag–O), 480 w ν(Ag–N), 429 w, 351 w, 267 m, 180 m. Raman (solid, cm^−1^): 2932 s, 1628 s, 1544 m, 1484 m, 1424 m, 1385 m, 1307 w, 1226 w, 1170 w, 1154 w, 1027 s ν_s_(SO_3_), 761 m, 707 w, 625 s ν(C–C), 593 m, 577 w, 523 w, 442 w, 353 m, 340 w, 319 m. ^1^H-NMR (CDCl_3_, 500 MHz): *δ*/ppm (assignments): 1.61 (s, br, 12 H, Me). UV–Vis (solution, MeOH, *λ*_max_/nm (*ε*/cm^−1^ mol^−1^ dm^3^)): 230 (shoulder, 8300). Emission (solution, MeOH, ex. 260 nm, λ_max_/nm): 337. Emission (solid, ex. 250 nm, λ_max_/nm): 83 K, 397; 173 K, 393; 298 K, 324.

### 3.4. X-ray Crystal Structure Determination

The diffraction data of **[Ag(CF_3_CO_2_)(Me_4_bpzH_2_)]** and **[Ag(CF_3_SO_3_)(Me_4_bpzH_2_)]** were obtained on a Rigaku XtaLAB P200 diffractometer using multi-layer mirror monochromated Mo K*α* (*λ* = 0.71073 Å) radiation at –95 ± 2 °C. A crystal of suitable size and quality was coated with Paratone-N oil (Hampton Research, Aliso Viejo, CA, USA) and mounted on a Dual-Thickness MicroLoop LD (200 μM) (MiTeGen, New York, NY, USA). The unit cell parameters were determined using *CrystalClear* from 18 images [37]. The crystal to detector distance was ca. 45 mm. Data were collected at 0.5° intervals in *φ* and *ω* to a maximum 2*θ* value of 55.0°. The highly redundant data sets were reduced using *CrysAlisPro* [38]. An empirical absorption correction was applied for each complex. Structures were solved by direct methods *(SIR2008* [39] and *SIR2004* [40]). The position of the silver ions and their first coordination sphere were located using a direct method (*E*-map). Other non-hydrogen atoms were found in alternating difference Fourier syntheses, and least squares refinement cycles. During the final refinement cycles, the temperature factors were refined anisotropically. Refinement was carried out by a full matrix least-squares method on *F*^2^. All calculations were performed with the *CrystalStructure* [41] crystallographic software package except for refinement, which was performed using *SHELXL 2013* [42]. Hydrogen atoms were placed in calculated positions. Crystallographic data and structure refinement parameters, including the final discrepancies (*R* and *Rw*), are listed in Table 1.

## 4. Conclusions

Silver(I) coordination polymers are important in coordination chemistry, but they often have very poor solubility in common solvents. To overcome this disadvantage, we synthesized silver(I) complexes with a trifluoromethyl group, viz **[Ag(CF_3_CO_2_)(Me_4_bpzH_2_)]** and **[Ag(CF_3_SO_3_)(Me_4_bpzH_2_)]**. We determined both solid-state structures at a low temperature. The Ag(I) atoms in **[Ag(CF_3_CO_2_)(Me_4_bpzH_2_)]** were coordinated by two pyrazole N atoms of two Me_4_bpzH_2_ and one O atom of a CF_3_CO_2_^−^ anion, giving a distorted trigonal pyramidal geometry. In the 1-D polynuclear structure, a zig-zag configuration was formed. Likewise, the coordinated CF_3_CO_2_^−^ anions were also located in a zig-zag pattern. By comparison, the Ag(I) atoms in **[Ag(CF_3_SO_3_)(Me_4_bpzH_2_)]** were coordinated by two pyrazole N atoms of two Me_4_bpzH_2_ and one O atom of the CF_3_SO_3_^−^ anion, giving a distorted trigonal pyramidal geometry. In the 1-D polynuclear structure, a linear configuration was formed. The coordinated anions CF_3_SO_3_^−^ were oriented in the same direction. This conformation was stabilized by two intermolecular hydrogen bonds, forming a double-chain structure. Solution properties were measured by ^1^H-NMR, UV–Vis absorption, and photoluminescence spectroscopies. These silver(I) coordination polymers exhibited interesting photoluminescence properties resulting from the presence of intermolecular argentophilic (Ag···Ag) interactions and/or ligand-based phosphorescence with the heavy atom effect. Further efforts to probe how the structures of coinage silver(I) coordination polymers are affected by ligand and coordination environments are in progress in our laboratory.

## Figures and Tables

**Table 1:** Crystal data and structure refinement of **[Ag(CF_3_SO_3_)(Me_4_bpzH_2_)]** and **[Ag(CF_3_CO_2_)(Me_4_bpzH_2_)]**.

Complex	[Ag(CF_3_CO_2_)(Me_4_bpzH_2_)]	[Ag(CF_3_SO_3_)(Me_4_bpzH_2_)]
CCDC number	2,227,168	2,227,169
Empirical formula	C_12_H_14_AgF_3_N_4_O_2_	C_11_H_14_AgF_3_N_4_O_3_S
Formula weight	411.13	447.18
Crystal system	Monoclinic	Triclinic
Space group	*P*2_1_/*n* (#14)	*P*$\bar{1}$ (#2)
*a*/Å	13.2724(2)	8.66665(15)
*b*/Å	8.67316(15)	9.91576(18)
*c*/Å	13.3047(2)	10.2913(2)
*α/*°	90	111.6690(19)
*β/*°	91.3059(17)	102.4538(17)
*γ/*°	90	90.8501(14)
*V*/Å^3^	1531.15(4)	798.20(3)
*Z*	4	2
*D*_calc_/g cm^−3^	1.783	1.860
*μ*(MoKα)/cm^−1^	13.560	14.391
2*θ* range, °	6–55	6–55
Reflections collected	23895	25699
Unique reflections	3516	3666
*R*_int_	0.0304	0.0270
Number of variables	199	208
Refls./Para. ratio	17.67	17.63
Residuals: *R*1 (*I* > 2 σ (*I*))	0.0337	0.0320
Residuals: *R* (All refl.)	0.0359	0.0351
Residuals: *wR2* (All refl.)	0.0999	0.0965
Goodness of fit ind.	1.054	1.078
Max/min peak,/e Å^−3^	1.27/–0.73	1.21/–0.45

^a^ *R* = Σ ||*Fo*| − |*Fc*||/Σ |*Fo* |; *wR2* = [(Σ (*w* (|*Fo*|^2^ − |*Fc*|^2^)^2^)/Σ *w* (*Fo*^2^))^2^]^1/2^.

## Data Availability

The crystallographic data are available from the Cambridge Crystallographic Data Centre (CCDC).

## Supplementary Materials

The following are available online at https://www.mdpi.com/article/10.3390/molecules28072936/s1, CIFs and check CIF reports. Figure S1: PXRD of **[Ag(CF_3_CO_2_)(Me_4_bpzH_2_)]** and the simulated diffractogram, Figure S2: PXRD of **[Ag(CF_3_SO_3_)(Me_4_bpzH_2_)]** and the simulated diffractogram, Figure S3: ^1^H-NMR spectrum of **[Ag(CF_3_SO_3_)(Me_4_bpzH_2_)]**, Figure S4: ^1^H-NMR spectrum of Me_4_bpzH_2_, Figure S5: UV spectra of the ligand and**[Ag(CF_3_SO_3_)(Me_4_bpzH_2_)]**. Figure S6: IR spectra of the ligand and silver(I) complexes, Figure S7: FT-Raman spectra of the ligand and silver(I) complexes, Figure S8: Photoluminescence spectra of the ligand and silver(I) complexes at 298 K, Figure S9: Temperature dependent photoluminescence spectra of the ligand Me_4_bpzH_2_, Figure S10: Temperature dependent photoluminescence spectra of **[Ag(CF_3_CO_2_)(Me_4_bpzH_2_)]**, Figure S11: Temperature dependent photoluminescence spectra of **[Ag(CF_3_SO_3_)(Me_4_bpzH_2_)]**, Figure S12: Solid-state photoluminescence spectra of the ligand and silver(I) complexes at 173 K, Figure S13: Solid-state photoluminescence spectra of the ligand and silver(I) complexes at 298 K.

## Sample Availability

Samples of the silver(I) compounds in this study are available from the authors.
