# Supplementary material for: Silver(I) Coordination Polymer Ligated by Bipyrazole Me4bpzH2, [Ag(X)(Me4bpzH2)] (X = CF3CO2− and CF3SO3−, Me4bpzH2 = 3,3′,5,5′-Tetramethyl-4,4′-bipyrazole): Anion Dependent Structures and Photoluminescence Properties
Source: Molecules. 2023 Mar 24;28(7):2936. doi: 10.3390/molecules28072936 (PMC10096423; doi:10.3390/molecules28072936)
Supplement: Supplementary file 1 [file molecules-28-02936-s001.zip › molecules-2235772-supplementary.pdf]

molecules
MDPI

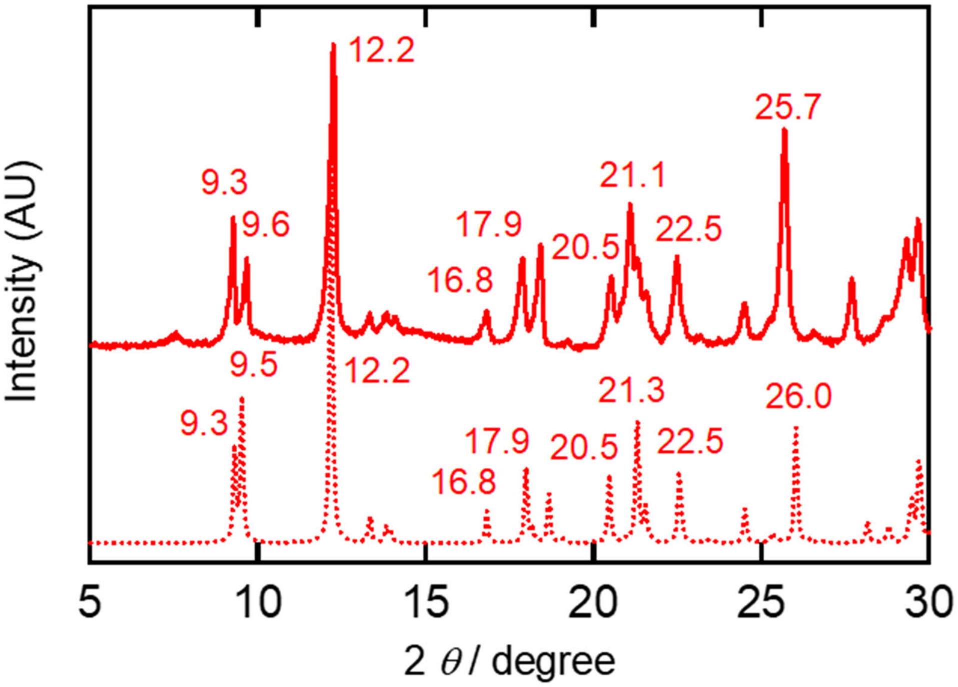

Figure S1. Powder X-ray diffractogram of **[Ag(CF_3CO_2)(Me_4bpzH_2)]** at room temperature. The simulated diffractogram (dashed line) is based on the X-ray data collected at –95 °C.

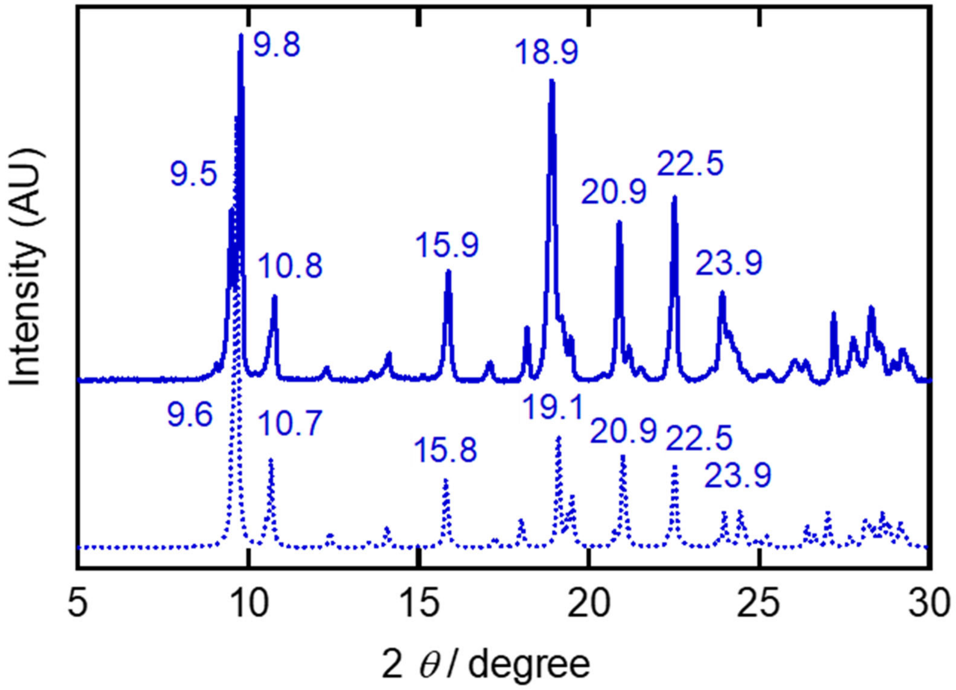

Figure S2. Powder X-ray diffractogram of **[Ag(CF_3SO_3)(Me_4bpzH_2)]** at room temperature. The simulated diffractogram (dashed line) is based on the X-ray data collected at –95 °C.

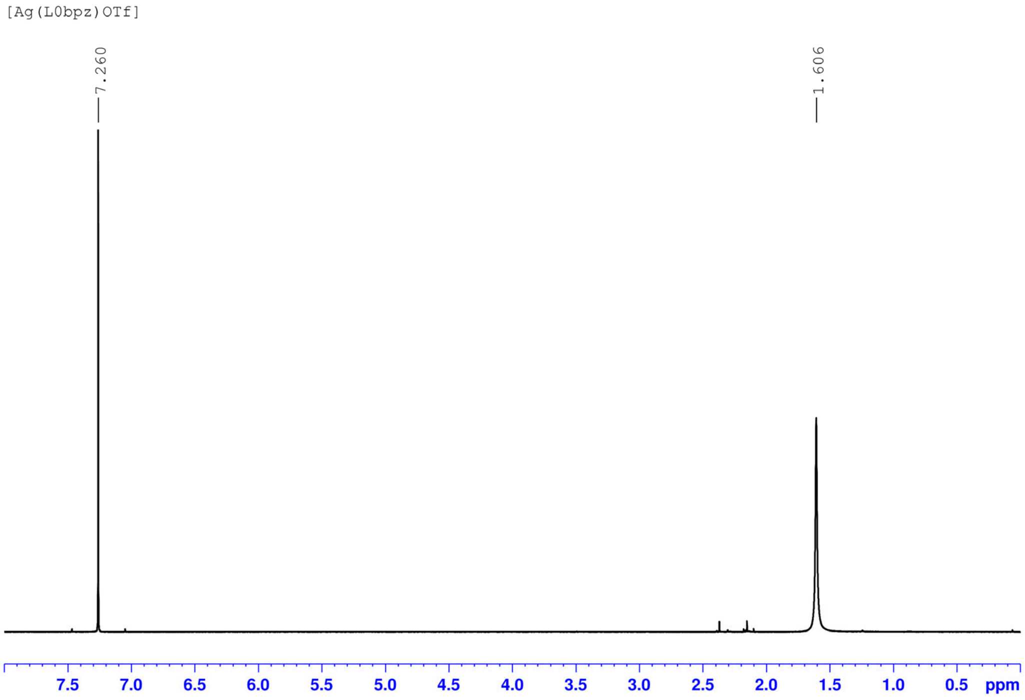

Figure S3. ^{1}H-NMR spectrum of **[Ag(CF_3SO_3)(Me_4bpzH_2)]** at room temperature in $CDCl_3$.

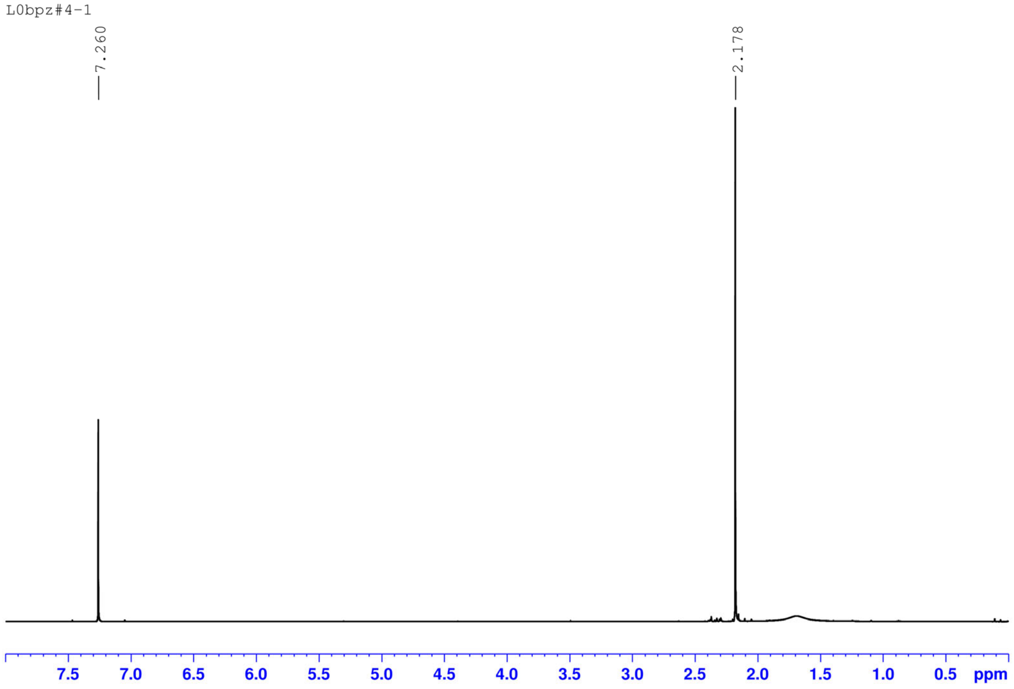

Figure S4. ^{1}H-NMR spectrum of Me_4bpzH_2 at room temperature in $CDCl_3$.

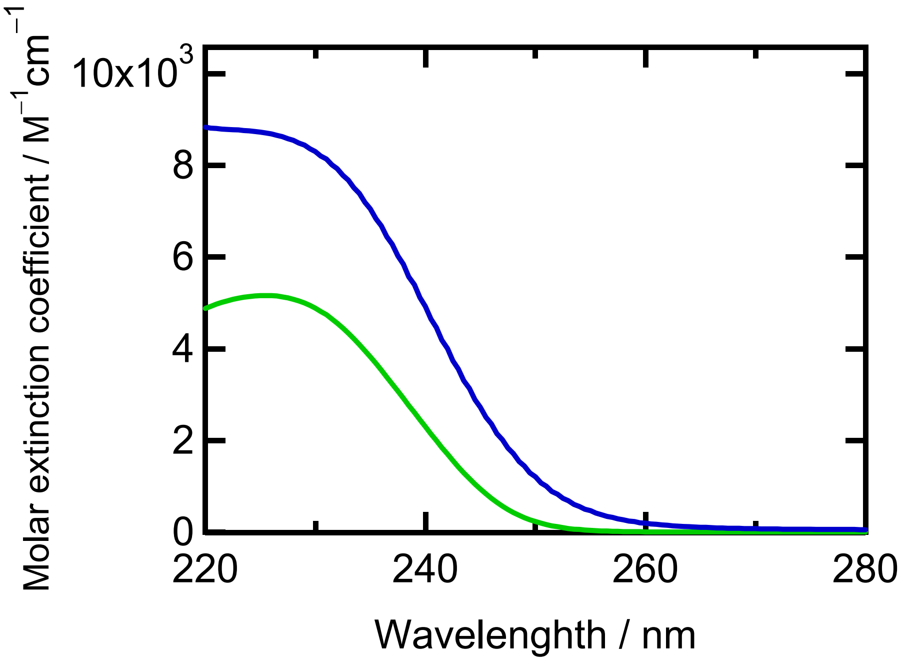

Figure S5. UV spectra of the ligand Me_4bpzH_2 (green line) and silver(I) coordination polymers **[$Ag(CF_3SO_3)(Me_4bpzH_2)$]** (blue line) at room temperature in MeOH.

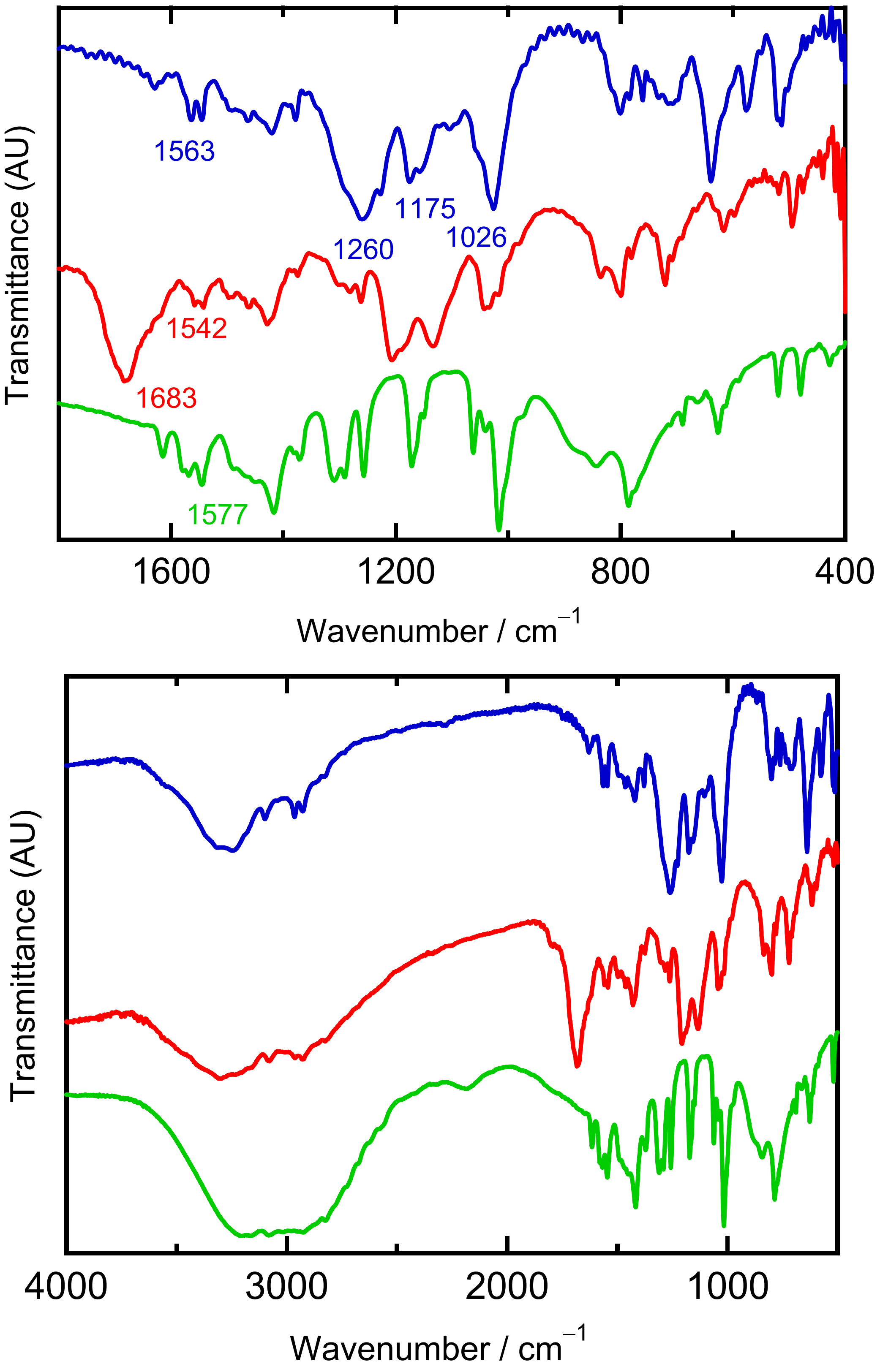

Figure S6. IR spectra of the ligand Me_4bpzH_2 (green line) and silver(I) complexes **[Ag(CF_3CO_2)(Me_4bpzH_2)]** (red line) and **[Ag(CF_3SO_3)(Me_4bpzH_2)]** (blue line) at room temperature. Top: 4000–500 cm^{-1} range, bottom: 1800–400 cm^{-1} region.

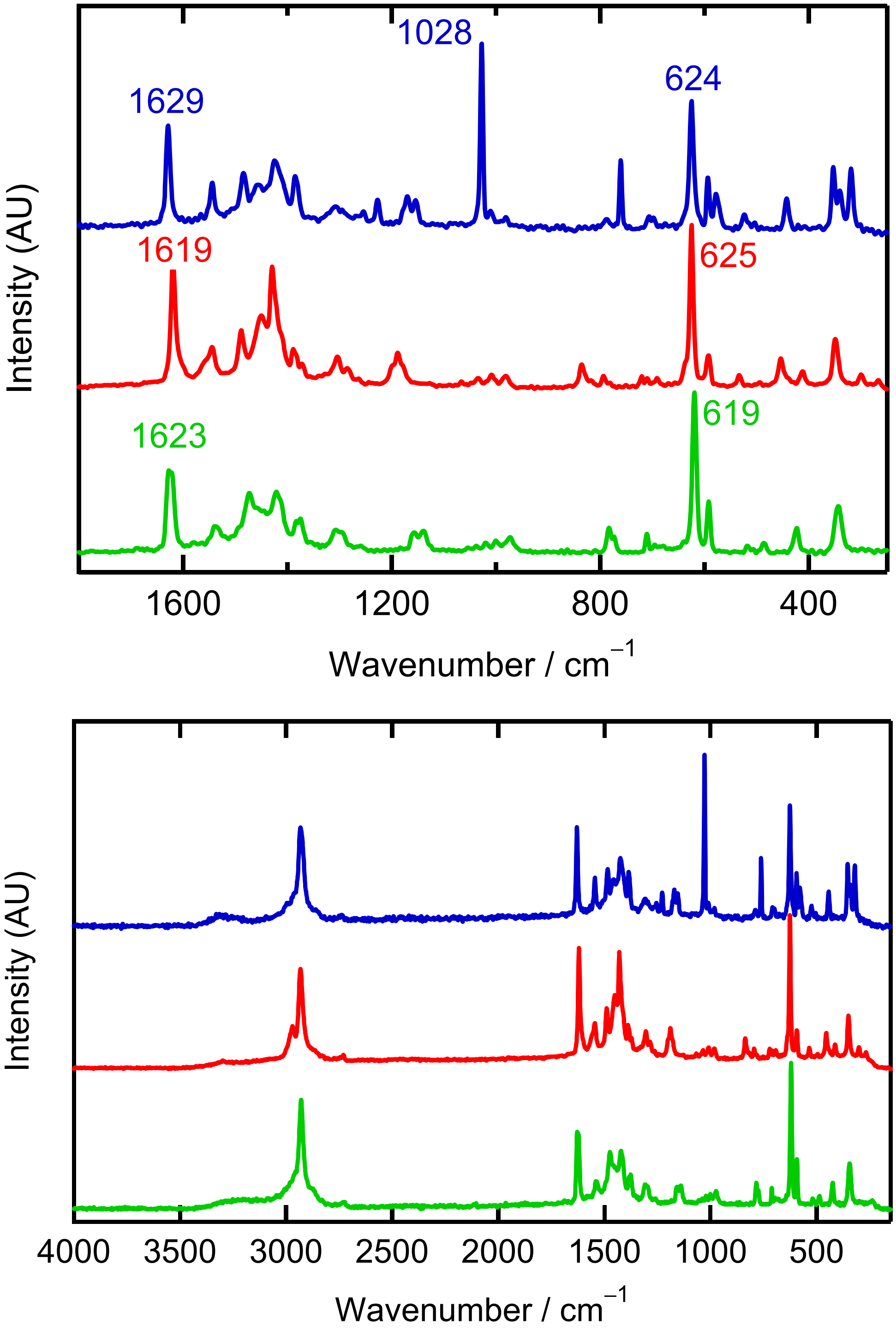

Figure S7. FT-Raman spectra (600 mW Laser power) of the ligand Me_4bpzH_2 (green line) and silver(I) complexes **[Ag(CF_3CO_2)(Me_4bpzH_2)]** (red line) and **[Ag(CF_3SO_3)(Me_4bpzH_2)]** (blue line) at room temperature. Top: 4000–150 cm^{-1} range, bottom: 1800–250 cm^{-1} region.

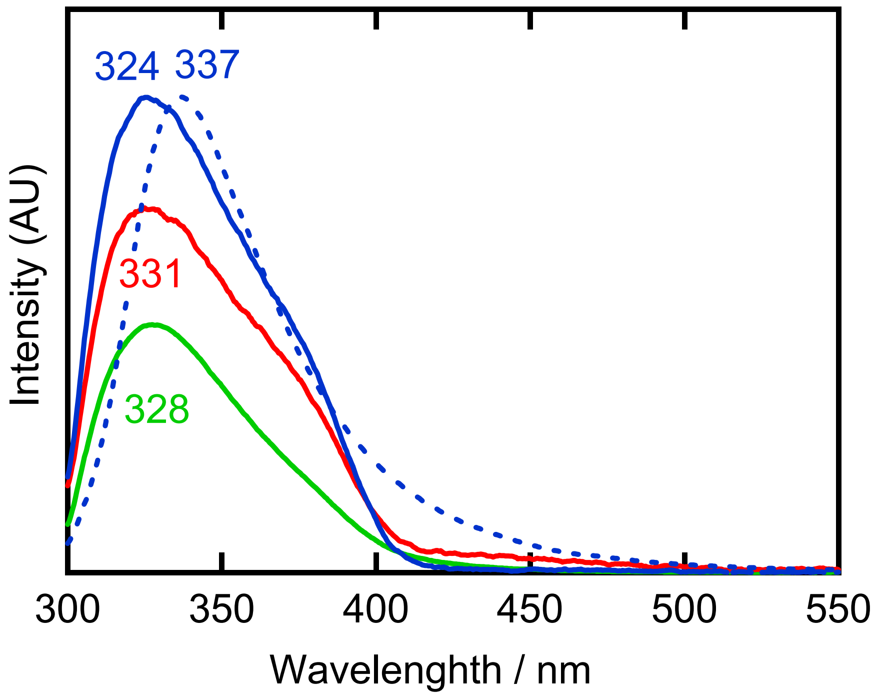

Figure S8. Photoluminescence spectra of the ligand Me_4bpzH_2 (solid-state, green line at 250 nm excitation), and silver(I) complexes **[Ag(CF_3CO_2)(Me_4bpzH_2)]** (solid-state, red line at 250 nm excitation) and **[Ag(CF_3SO_3)(Me_4bpzH_2)]** (solid-state, blue line at 240 nm excitation and solution-state in MeOH, blue dashed line at 260 nm exaction) at 298 K.

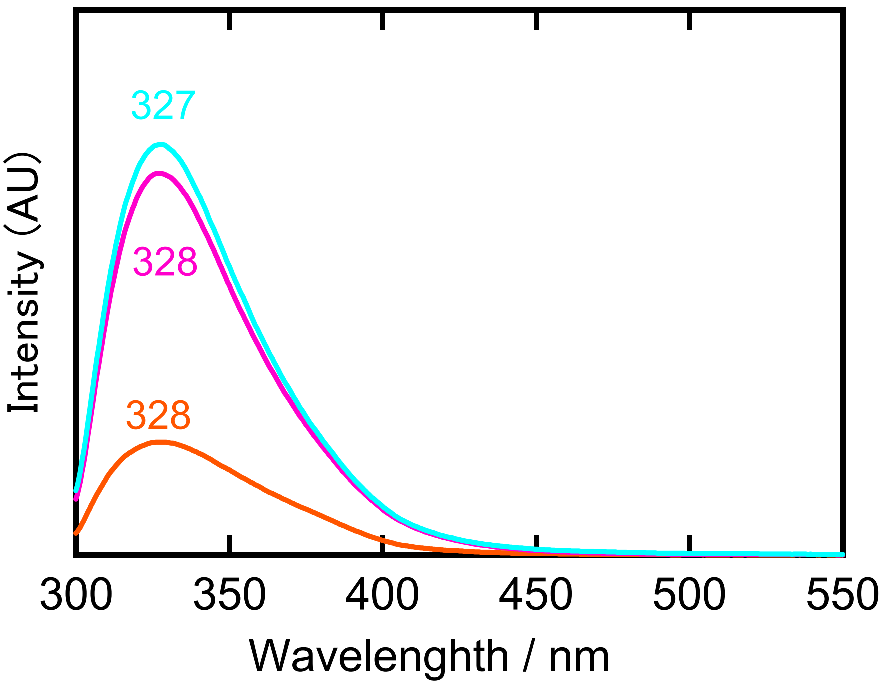

Figure S9. Temperature dependent photoluminescence spectra of the ligand Me_4bpzH_2 (solid-state) at 250 nm excitation wavelength, temperatures: (83 K (light blue), 173 K (purple), and 298 K (orange).

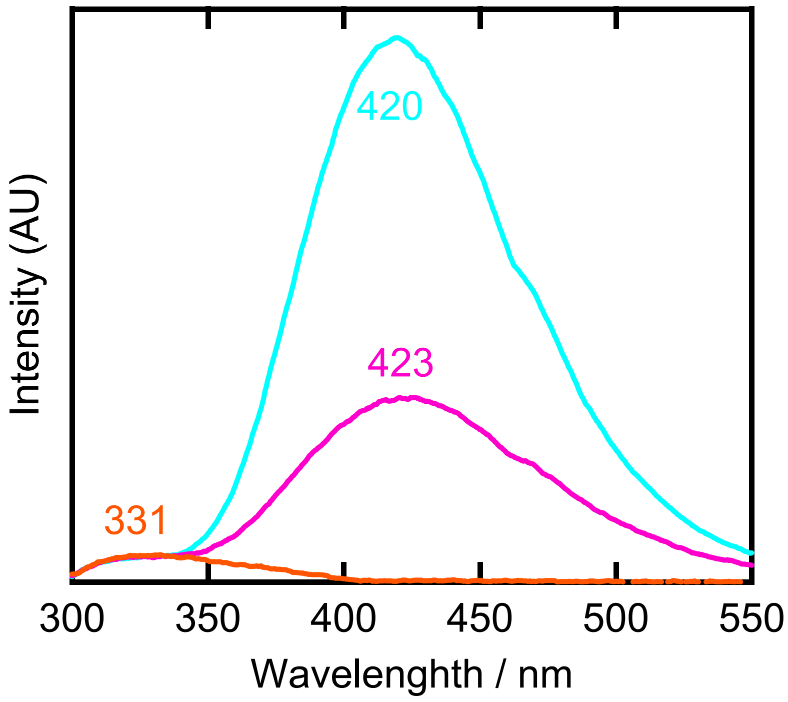

Figure S10. Temperature dependent photoluminescence spectra of **[Ag(CF_3CO_2)(Me_4bpzH_2)]** (solid-state) at 250 nm excitation wavelength, (83 K (light blue), 173 K (purple), and 298 K (orange).

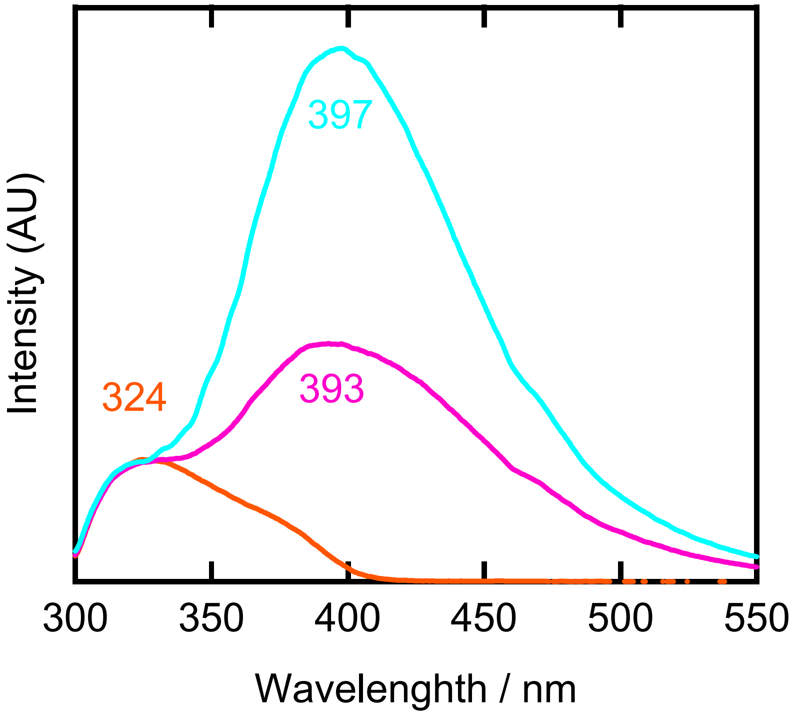

Figure S11. Temperature dependent photoluminescence spectra of **[Ag(CF_3SO_3)(Me_4bpzH_2)]** (solid-state) at 240 nm excitation wavelength, (83 K (light blue), 173 K (purple), and 298 K (orange).

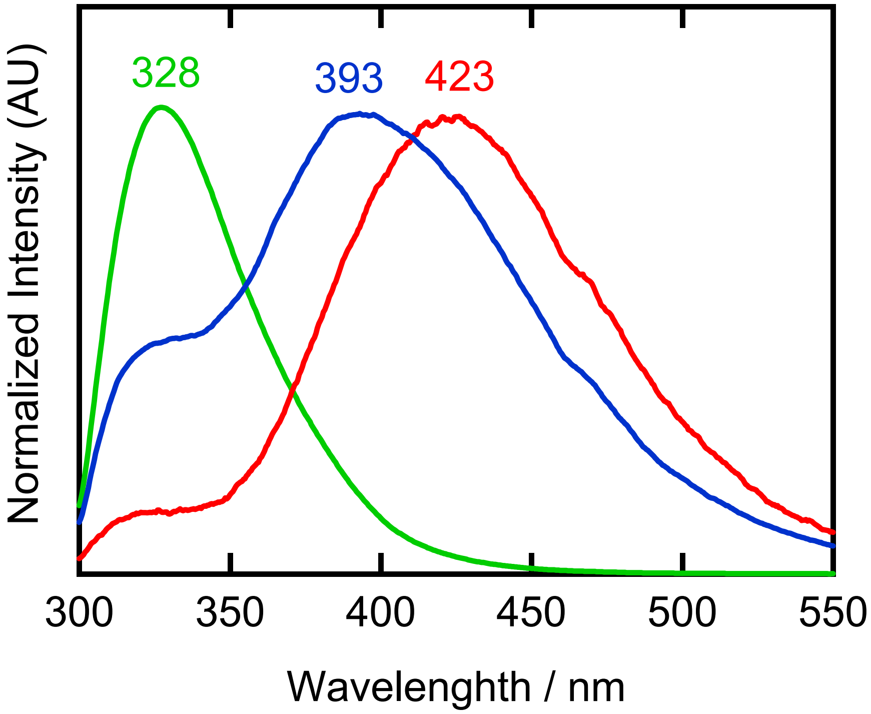

Figure S12. Solid-state photoluminescence spectra of the ligand Me_4bpzH_2 (green line, 250 nm excitation) and silver(I) complexes **[Ag(CF_3CO_2)(Me_4bpzH_2)]** (red line, 250 nm excitation) and **[Ag(CF_3SO_3)(Me_4bpzH_2)]** (blue line, 240 nm excitation) at 173 K

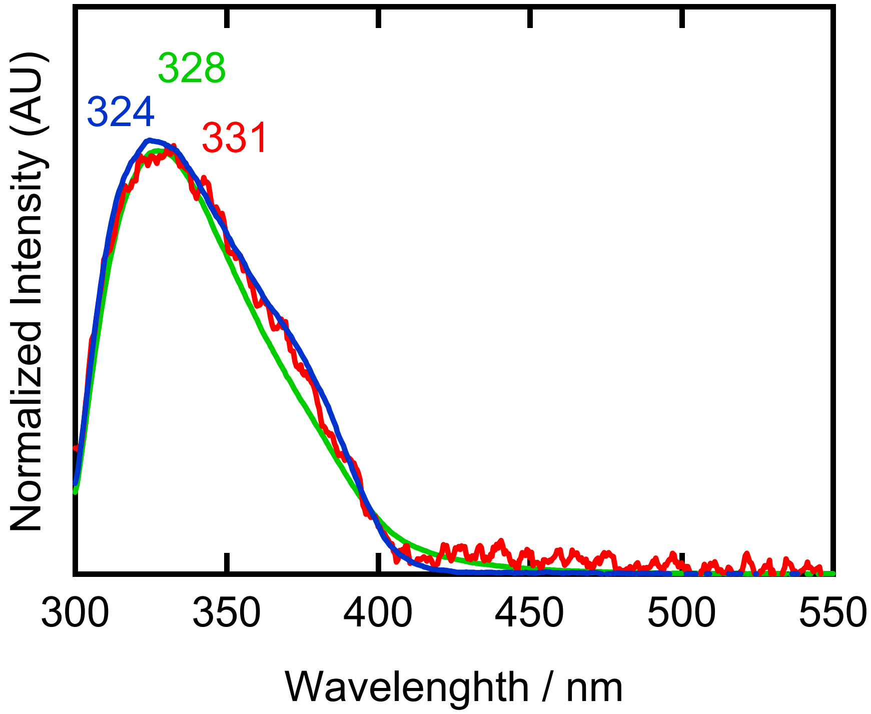

Figure S13. Solid-state photoluminescence spectra of the ligand Me_4bpzH_2 (green line, 250 nm excitation) and silver(I) complexes **[Ag(CF_3CO_2)(Me_4bpzH_2)]** (red line, 250 nm excitation) and **[Ag(CF_3SO_3)(Me_4bpzH_2)]** (blue line, 240 nm excitation) at 298 K.
